# *In vivo* study of non-invasive effects of non-thermal plasma in pressure ulcer treatment

**DOI:** 10.1038/s41598-018-24049-z

**Published:** 2018-04-04

**Authors:** Maedeh Chatraie, Giti Torkaman, Mohammadreza Khani, Hossein Salehi, Babak Shokri

**Affiliations:** 1 Laser applications in medical sciences research center, Shahid Beheshti University of Medical Sciences, Tehran, Iran; 2 Laser - Plasma Research Institute, Shahid Beheshti University, G.C., P.O. Box, 19839-6941 Tehran, Iran; 3 Physics Department of Shahid Beheshti University, G.C., P.O. Box, 19839-6941 Tehran, Iran; 4 Physical Therapy Department, Faculty of Medical Sciences, Tarbiat Modares University, Tehran, Iran; 5 Department of Anatomical Sciences, School of Medicine, Isfahan University of Medical Sciences, Isfahan, Iran

## Abstract

According to high incidence and prevalence of pressure ulcers worldwide, the purpose of this study is using of non-thermal atmospheric plasma as a novel therapy for pressure ulcers. Cold plasma was produced by applying a high-voltage (5 kV) and high-frequency (25 kHz), to helium gas. Under general anesthesia and sterile conditions, two circular magnets were used to create pressure ulcers on the dorsal skin of adult rats. The wounds were divided randomly into control and plasma-treated groups. Animals in the plasma-treated group received plasma radiation for 5 days, each day 3 times and every time 60 s. Mechanical assays were performed to determine plasma effects on the mechanical strength of the repaired tissue. The results showed that mechanical strength of repaired wound in the plasma-treated group was significantly higher than that in the control group (p < 0.05). In addition, evidence from histological studies indicates a significantly accelerated wound re-epithelialization in comparison with the control group; angiogenesis and fibrosis (collagen synthesis) were also significantly increased and the inflammation phase of wound healing was shorter in the plasma-treated group. The plasma treatment also resulted in significant wound contraction and acceleration of wound healing. The findings of present study indicate the effects of cold plasma on pressure ulcer treatment.

## Introduction

Pressure ulcer (also described as bed sore and decubitus ulcer) is a major health challenge worldwide that imposes a significant financial burden on health care systems and negatively affects quality of life, so quick and opportune curative actions are needed^1^. Definition of pressure ulcer provided by the National Pressure Ulcer Advisory Panel is “localized injury to the skin and/or underlying tissue usually over a bony prominence, as a result of pressure or pressure in combination with shear”^2,3^. The main groups of people at risk of developing pressure ulcers include elderly people over 70 years, patients with spinal cord injuries, hospitalized patients, especially those undergoing orthopedic surgeries and patients in especial care^2–6^. According to the epidemiological studies, approximately 40% of patients with spinal cord injury experience a pressure ulcer^7,8^.

The current care strategies of pressure ulcers comprise surgical debridement of all necrotic tissue, negative pressure wound therapy, maintenance of a favorable moist wound bed by employing suitable dressings, relief of pressure and verifying issues such as nutritional, metabolic and circulatory status^9,10^. The consequences of pressure ulcers that afflict patients and health care systems, justify the requirement of extensive efforts to reduce this problem. Using of non-thermal atmospheric pressure plasma, as a possible safe new method in chronic wound therapy has been reported in many different studies^11–20^.

Plasma is defined as a partially or completely ionized medium that is composed of many elements such as charged particles (electrons, ions), neutral and excited atoms, UV photons and radicals^21–24^. In recent years the application of non-thermal plasma in biomedical researches such as wound healing, blood coagulation^23^, angiogenesis suppression^25^, cancer treatment^26,27^ and deactivation of microorganisms^28,29^ like MDR bacteria^30^ and even viruses^31^ is rapidly growing, primarily due to its bactericidal properties. A large volume of studies have identified the positive effects of non-thermal plasma on healing acne^32^ and eczema^33^, venous ulcers^34–36^, chronic leg ulcers^17,37^, burn wounds^38,39^, full-thickness cutaneous wounds in healthy and diabetic mice^19,40^, and skin rejuvenation^41^. Unfortunately, the main mechanism of the non-thermal plasma interaction with cells or living tissue still remains unknown^23^, but the effects of non-thermal plasma on the key wound-related cells have been reported in several studies. These studies have shown that plasma induces fibroblast migration and proliferation^42^, promotes the proliferation of endothelial^43^ and keratinocyte^14^ cells, as well as the growth of epithelial cells^44^; these effects are attributed to the production of intracellular reactive oxygen species (ROS)^45^. In addition, NO-containing plasma induces skin acidification (pH reduction) as a result of NO_x_ interaction with water and increases dermal microcirculation^46^. These effects are similar to those found in the natural process of wound healing. Therefore, plasma therapy represents a promising new medication against chronic/infected wounds.

To our knowledge, this is the first study that reports the effects of cold plasma on the treatment of an animal model of pressure ulcer. In order to compare the wound healing process between plasma-treated and control specimens, mechanical and histological parameters were measured and wound contraction was evaluated during the healing period.

## Results

### Wound area reduction

**Figure 1:**
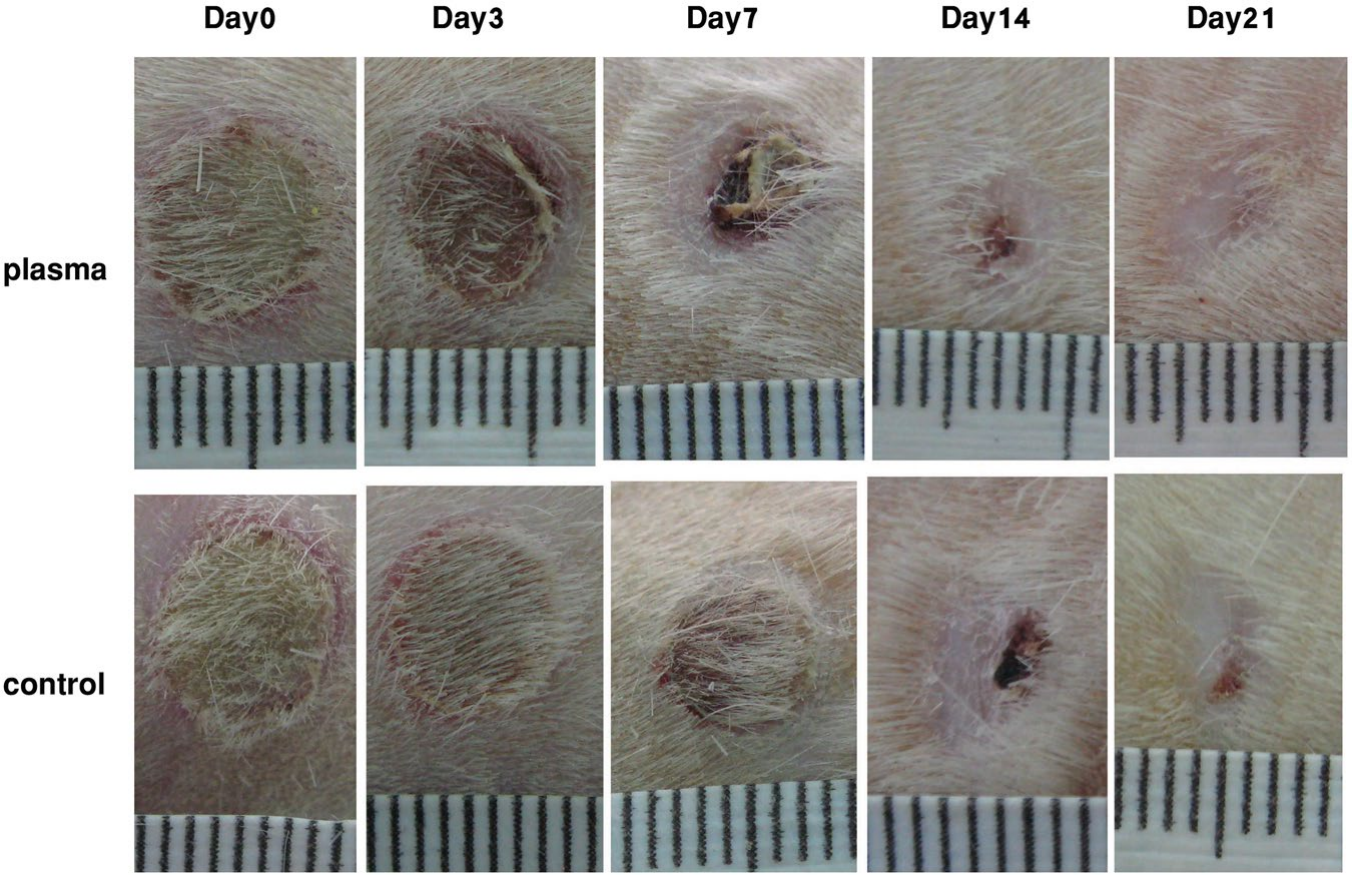
Macroscopic observation of wound healing on different days of healing period.

Wounds were observed and evaluated daily from day 0 to complete healing. As shown in Fig. 1 there were no particular differences in the appearance of the wound surfaces between two groups, except in the wound size in several days of the healing period. Wound size reduction was observed in both control and plasma-treated groups, but from Day 7 to Day 21 the wound size in the plasma-treated group was significantly smaller than that in the control group. Wound healing duration in the plasma-treated group was 17.83 ± 0.83 days whereas in the control group after Day 21 some wounds didn’t heal completely yet and wound healing duration was about 19.37 ± 0.62 days.

**Figure 2:**
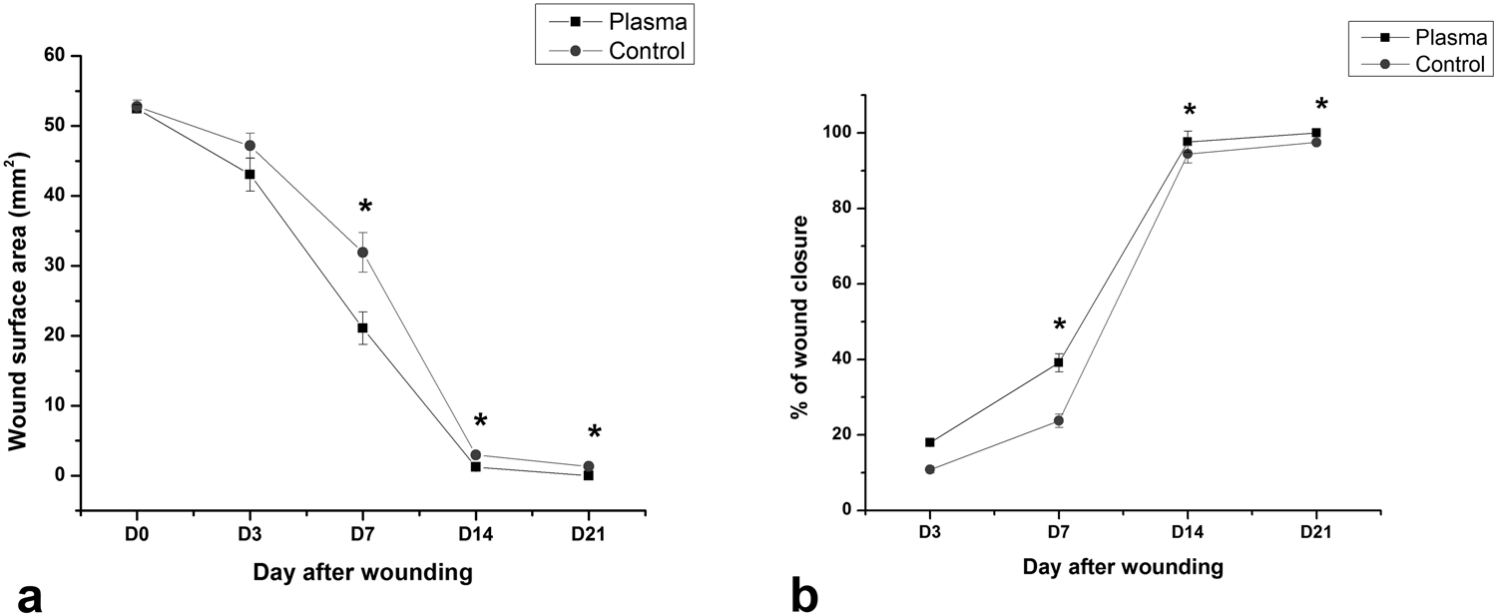
(**a**) Wound area measurement. (**b**) Percentage decrease of wound surface area on different days of healing period. The treated wound was significantly smaller than that of the control (mean ± SEM, *p < 0.05).

The wound area and the percentage decrease in wound size, in all animals were calculated and reported in Fig. 2(a,b), respectively. According to these Figures, there are no significant differences in wound area between plasma-treated and control groups on days 0 and 3 after wounding. On days 7–21, wound area surfaces in the plasma-treated group are significantly smaller than those in the control group and so the percentage decrease of wound area is significantly higher in the plasma-treated group (p < 0.05). F value for the wound area data on days 14 and 21 post wounding was obtained 10.16 and 13.09 respectively, and 13.82 for the percentage decrease in wound size on day 21.

### Effect of non-thermal plasma on histological and mechanical parameters

**Figure 3:**
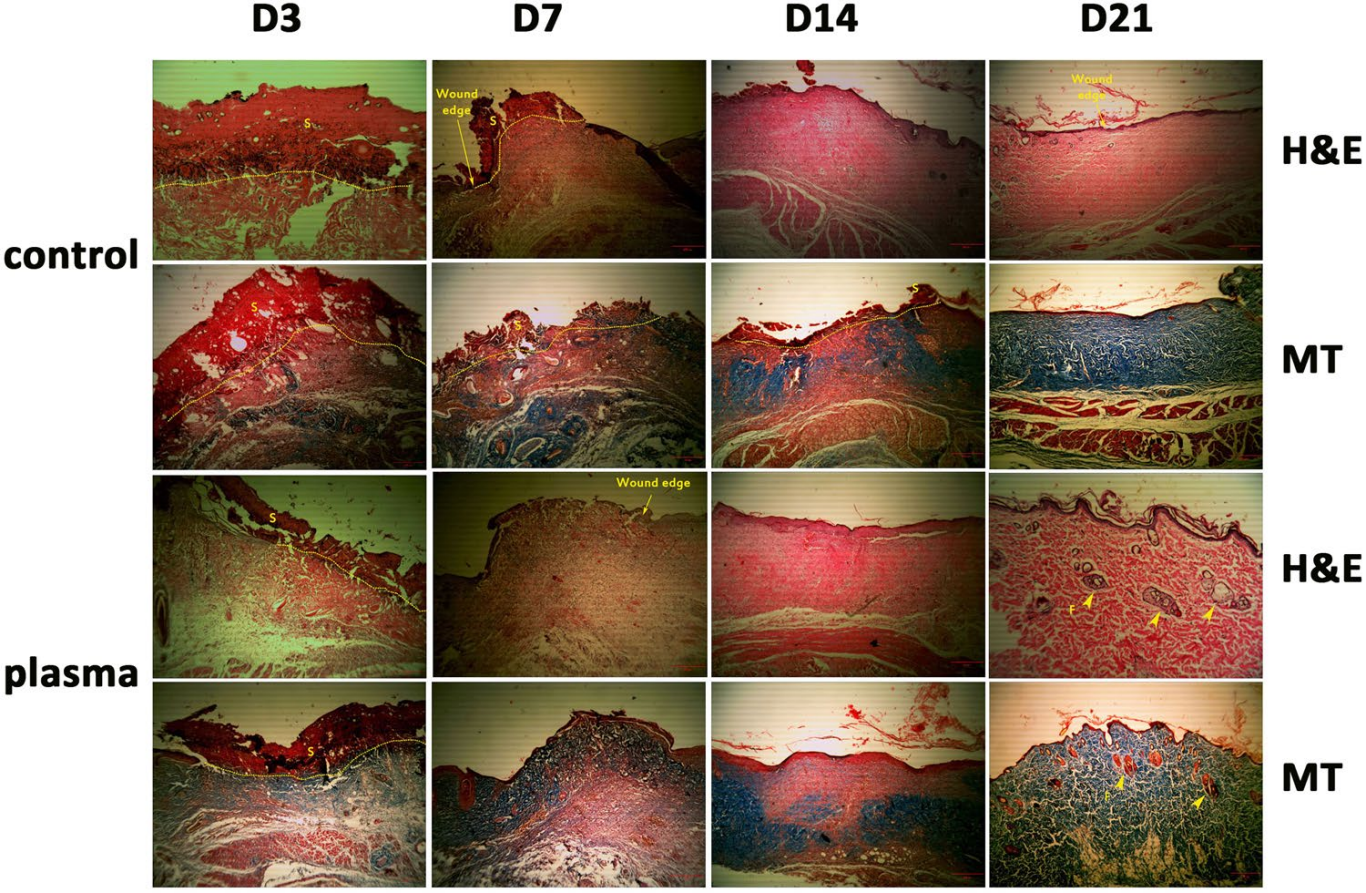
Microscopic images of H&E and Masson’s trichrome (MT) stained histological sections of control and plasma treated wounds on different days of healing period. Wound edge indicates the excision rim. S, scare; F, follicle; Scale bars, 200 μm.

Histological microscopic images from the tissue samples prepared on 3, 7, 14, and 21 days after wounding are presented in Fig. 3. It was observed that at day 3 after wounding: a new epidermal layer formed in plasma-treated animals which did not appear in control rats; production of scar tissue in control wounds was more, and less collagen generation happened in this group in comparison with the plasma-treated samples. Angiogenesis was not observed and infiltration of inflammatory cells was severe in both groups, indicating that immune defense increased in the early phases of wound healing.

At day 7: in the plasma-treated group, the epidermis began to regenerate and local presence of epidermis can be observed. In addition, unlike to the control group, production of scar tissue was stopped at this stage. Moreover, according to Masson’s trichrome staining, plasma caused stimulation of collagen synthesis in the dermis layer. The angiogenesis in the two groups increased from day 3 to 7, but it can be demonstrated that more angiogenesis was induced by plasma treatment.

At day 14: unlike to the control group, a thickened epidermis layer which covered the wound surface completely can be observed in the lesions treated with plasma. Moreover, angiogenesis was significantly increased because of plasma treatment. The inflammation decreased in both groups from day 7 to 14, but plasma caused a more rapidly reduction of inflammation in comparison with the control group.

At day 21: a new epidermis layer was covered the whole surface of all wounds, but in the plasma-treated wounds the epidermis was significantly thicker compared to the control group. The most important point obtained from the images is the formation of the new hair follicle and sebaceous glands in the epidermis and dermis layer of plasma-treated samples which did not appear in control rats. In addition, no inflammation was observed after plasma treatment while it cannot be ignored in control self-healed lesions.

**Figure 4:**
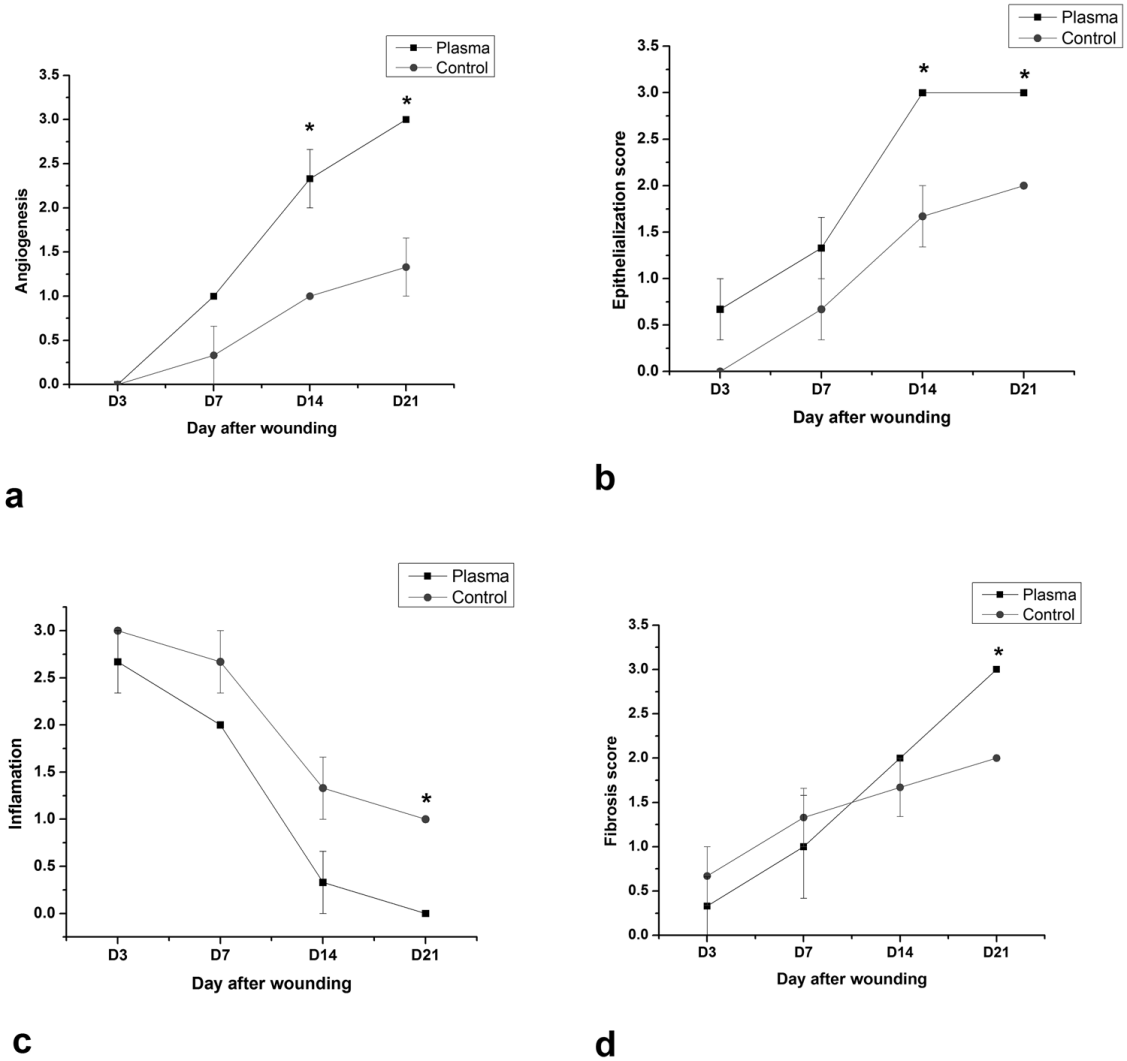
Histological scoring of (**a**) epithelialization, (**b**) inflammation, (**c**) collagen synthesis, and (**d**) angiogenesis during the healing period days. (mean ± SEM, *p < 0.05).

The results of statistical analysis of histopathological studies are shown in Fig. 4(a–d). According to data, significant differences in epithelialization, inflammation, collagen synthesis and angiogenesis were observed between groups (p < 0.05). In the plasma-treated group, epithelialization and angiogenesis were significantly more than that of the control group on days 14 and 21. On the other hand, after plasma treatment, the inflammation was significantly less than that of the control group on day21. At the same time in the plasma-treated group, there was significantly more collagen synthesis in comparison with that of the control group (p < 0.05).

### Maximum force and stress

**Figure 5:**
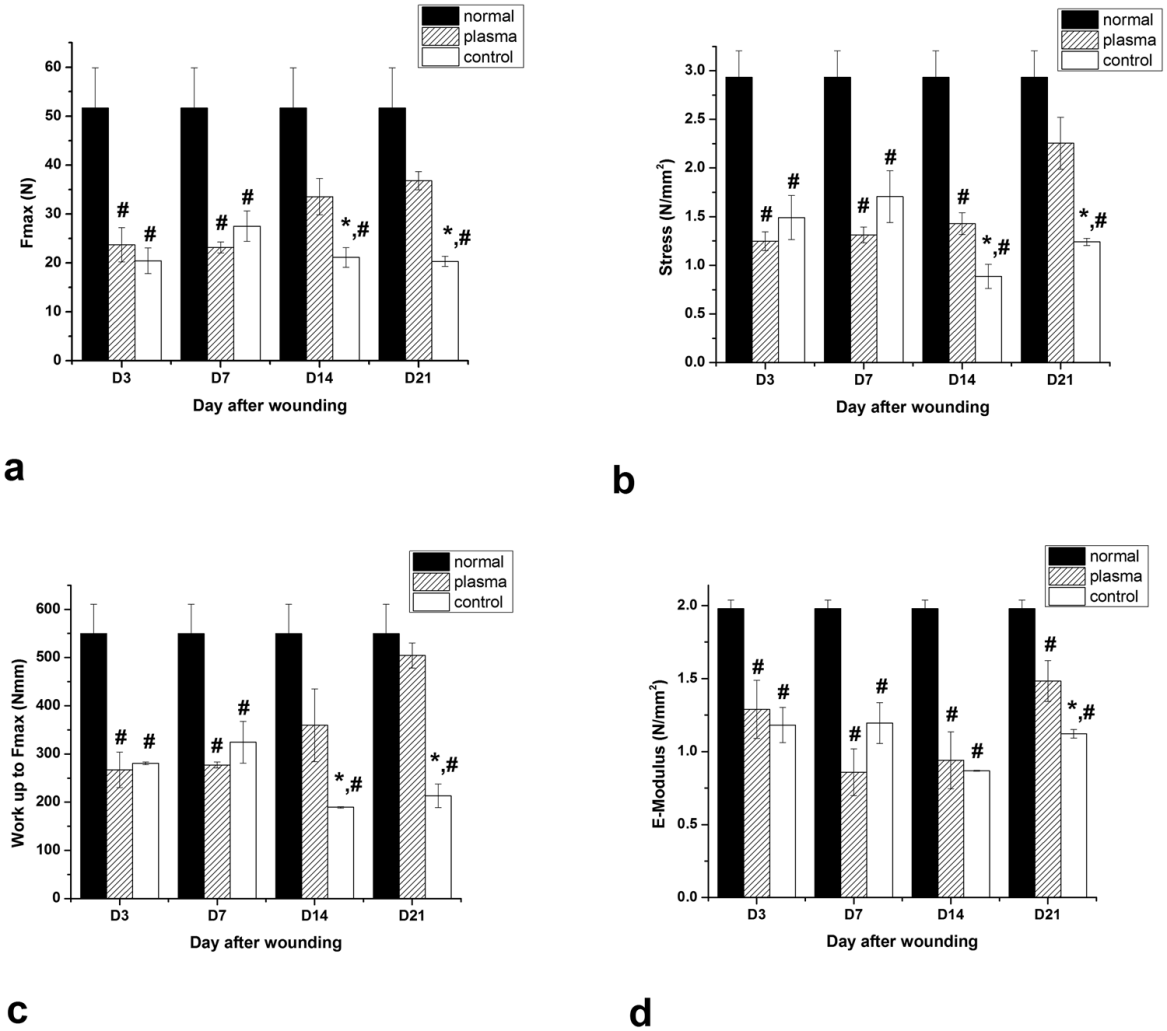
Comparing of (**a**) maximum force, (**b**) maximum stress, (**c**) work up to maximum force and (**d**) elastic stiffness between different groups during healing period (mean ± SEM). *Shows significant difference compared with plasma group and ^#^compared with normal skin (p < 0.05).

As exhibited in Fig. 5(a,b), the maximum force (F_max_) and maximum stress (maximum force per mm^2^), on days 3 and 7 after wounding decreased significantly (decreasing of tissue tolerance versus rupture) in both plasma-treated and control groups compared to normal skin because of tissue injury and tensile strength reduction. On days 14 and 21, a significant increase in maximum force and maximum stress was observed in the plasma-treated group compared to the control group, which showed a higher tensile strength of plasma- treated wounds (p < 0.05). The amount of maximum force and maximum stress in plasma-treated and control groups were always lower than those in normal skin but on days 14 and 21, the means value in the plasma-treated group were not significantly different from normal skin(p > 0.05), that means cold plasma radiation increases the tolerance of the repaired tissue against rupture.

### Work up to maximum force (W up to F_max_)

W up to F_max_ is the area under the Stress-Strain curve and represents energy absorption by the tissue under the tensile force applying. As shown in Fig. 5(c), similarly to maximum force and maximum stress, W up to F_max_ on days 3 and 7 decreased significantly in both plasma-treated and control groups compared to normal skin (p < 0.05). In the other hand on days 14 and 21, W in the plasma-treated group increased significantly compared to the control group but there was no significant difference between normal skin and plasma-treated wounds (p > 0.05). The increase of work up to maximum force in the plasma-treated group compared to the control group can be attributed to more regeneration of tissue in plasma-treated sores.

### Elastic Stiffness (E-Modulus-N/mm^2^)

Elasticity module (E-Modulus) is the slope of the linear part of the Stress-Strain curve and represents the elastic stiffness of tissue that is a very important parameter in the evaluation of wound healing process. Due to wounding, the stiffness and elasticity of tissue were significantly decreased so the skin was loosed. As shown in Fig. 5(d), E-Modulus in both plasma-treated and control groups was significantly lower than that in normal skin in all days of healing period, but the means in plasma-treated specimens were more than control and finally on day 21, E-Modulus increasing in the plasma-treated group was significant compared to the control group (p < 0.05). The increasing of the elastic stiffness of the plasma-treated wounds indicated that those tissues gained more elasticity during the repairing period. According to this fact that collagen fibers are responsible for the stiffness and elasticity of the skin tissue, increased elastic stiffness in plasma-treated wounds can be due to increased collagen synthesis in those tissues, as confirmed by the results of the histological test.

### Characterization of the He plasma jet

#### Optical emission spectroscopy

**Figure 6:**
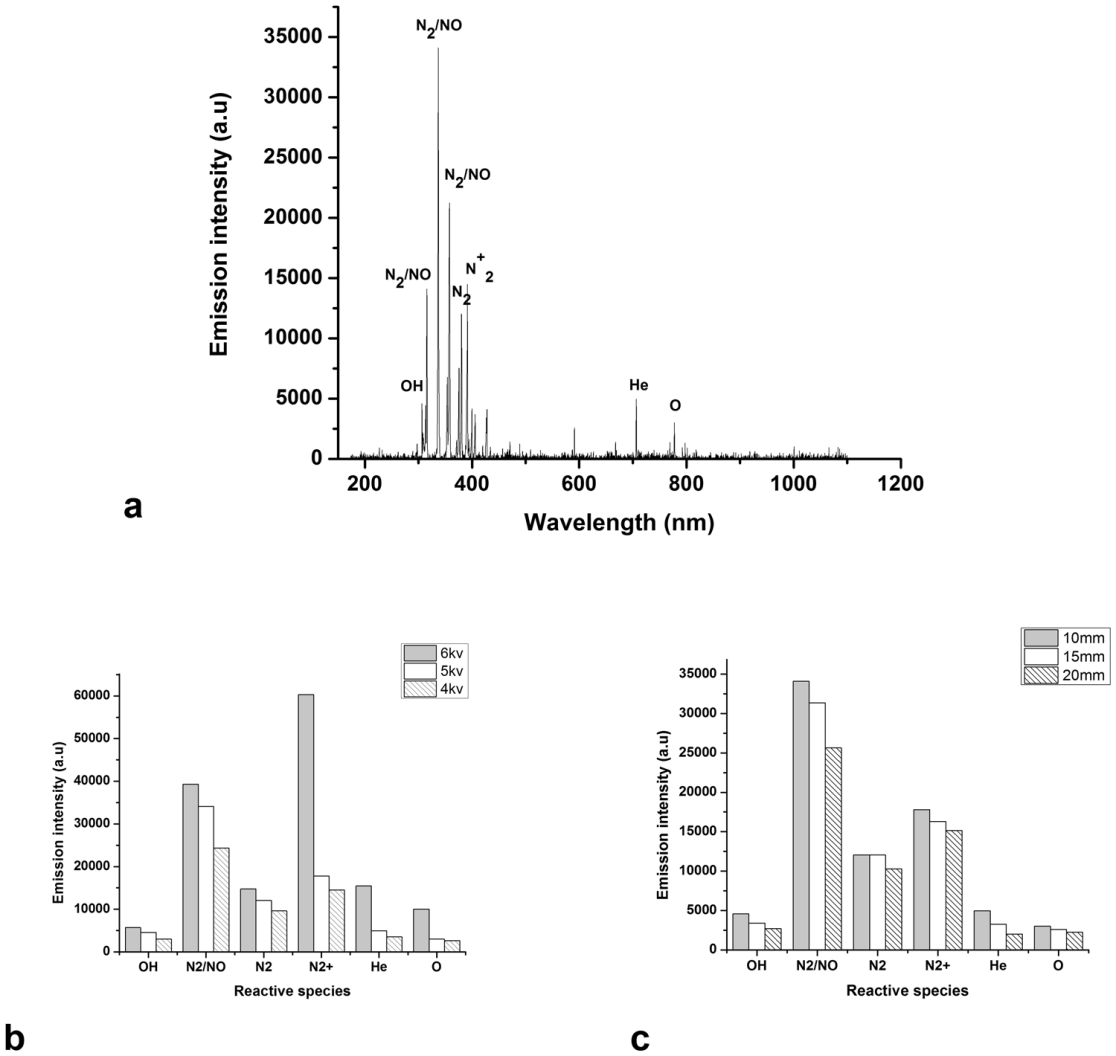
(**a**) Optical emission spectra of He plasma jet and excited species produced by it (Gas flow rate 4 slpm, peak to peak voltage = 5 kv, d = 10 mm distance from the nozzle). (**b**) Reactive species concentration against distance from the nozzle at 5 kV. (**c**) Reactive species concentration against peak-to-peak voltage at 10 mm distance from the nozzle.

Our results of the optical emission spectroscopy of the helium plasma jet were in agreement with other findings according to the literature^47^. According to Fig. 6(a), OES measurement at the distance of 10 mm under the nozzle and pick-to-pick voltage of 5 kv, showed the emission of species such as OH radical (306 nm), N_2_ or NO lines (316, 337, 358, 427.5 nm), N_2_^+^ (391 nm), He (706 nm), O (777 nm), NO (297 nm) and N_2_ lines(351.8, 353.6, 357.6, 375.4, 380, 399.7, 406 nm).

In this work, the effects of voltage and distance from the nozzle on the concentration of reactive species were studied. As shown in Fig. 6(b,c), by increasing the distance from the nozzle and the peak-to-peak voltage, the concentration of reactive species decreases and increases respectively.

#### Plasma temperature approximation

**Figure 7:**
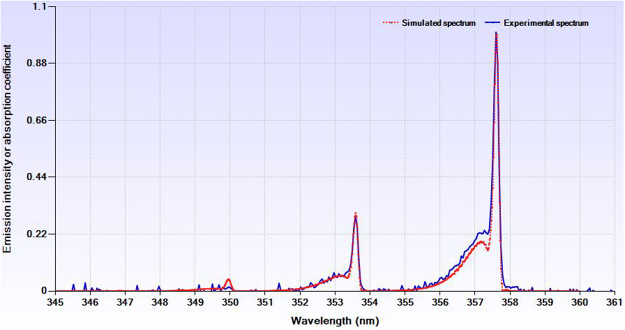
Comparing simulated and experimental spectra.

The rotational and vibrational temperatures can be approximated through comparing experimental and simulated spectra. As shown in Fig. 7, the best fitting was obtained at the rotational temperature of 300 k and the vibrational temperature of 3900 k.

#### Mouse skin temperature versus voltage

**Figure 8:**
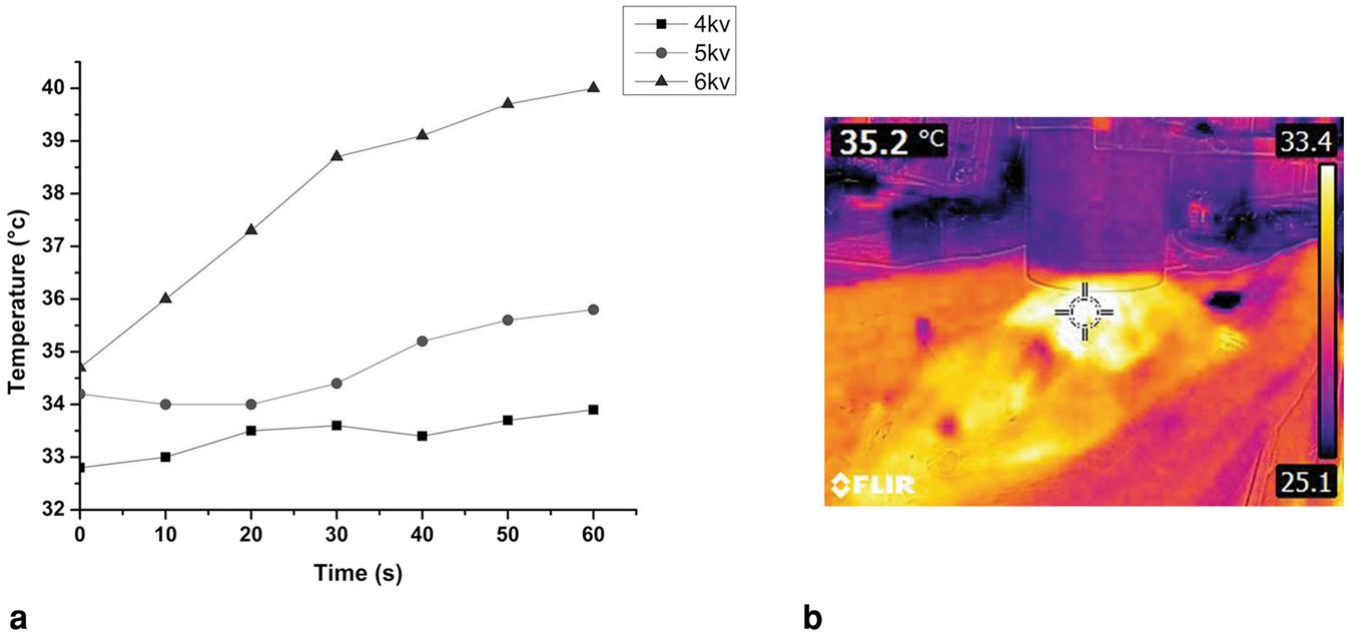
(**a**) Variation of temperature during time at different voltages. (**b**) Infrared thermal image at 5 kV and 60 s plasma treatment.

The results of temperature measurement in different voltages during 1 min plasma exposure were shown in Fig. 8(a). At the constant distance of 10 mm, by increasing the peak-to-peak voltage, the tissue temperature increases, it is because of increasing the electron energy and ionization rate in higher voltages. Plasma treatment with voltages of 4, 5 and 6 kv caused Δ*T* (elevated normal skin temperature) of about 1, 1.4 and 5 °C respectively. Δ*T* at 4 kv was lower than those at 5 and 6 kv, but according to the weak emission intensity of reactive species at 4 kv it’s not the optimum voltage and 5 kv was considered as the optimum. Figure 8(b) represents an infrared thermal image of normal and unwounded skin under plasma exposure at 60 s.

## Discussion

The results of the present study showed that cold plasma as a safe treatment method can positively affect the healing process of pressure sores. In this study after plasma characterization to find the optimal power and temperature of plasma, by controlling the input voltage and the distance from the tip nozzle, we focused on the changes of mechanical and histological parameters of treated tissue.

The results showed that plasma treatment accelerated the wound healing process in contrast to self-healed wounds. In addition, histological analysis revealed plasma supported effective re-epithelialization, angiogenesis, the formation of a new hair follicle and collagen fibers, and controlling of inflammation. Moreover, the mechanical analysis demonstrated that plasma can improve the mechanical strength and tissue tolerance versus uniaxial tensile load.

It’s believed that reactive oxygen and nitrogen species (ROS and NOS) generates in plasma play an important role in the plasma and tissue interaction. When the discharge gas entered the ambient air, the collisions and interactions of energetic and ionized helium molecules with the surrounding air, led to the ionization of nitrogen and oxygen molecules; so, ROS and NOS can be observed in helium plasma spectrum. By increasing the distance from the nozzle, the electrical field decreases and both the electron density and energy are too low to happen ionization and excitation. Therefore, the concentration and intensity of reactive species in this region is lower. In the other hand, more ionization happens in higher voltages and it results in increasing reactive species production.

By analyzing the histological microscopic images, it can be obtained that plasma treatment is significantly effective for the acceleration of wound re-epithelialization and angiogenesis. In confirmation of our findings, similar results were reported in previous studies. Nasruddin *et al*.^18^, and M.-H. Ngo Thi *et al*.^38^, reported that cold plasma accelerated the re-epithelialization of full-thickness and burn wounds respectively. Furthermore, the influence of plasma on angiogenesis acceleration as an important physiological process in wound healing has been reported by Haertel *et al*.^48^. It was hypothesized that ROS and RNS produced in the plasma, induced the angiogenesis and epithelialization processes through enhancing cell proliferation and migration by ROS/RNS signals^38,49^. Furthermore, in several studies, it was reported that topical application of NO significantly accelerated wound healing, followed by less inflammation, promoted re-epithelialization, and more angiogenesis^50,51^. Therefore NO-containing plasma can be suggested as a promising strategy for controlling wound inflammation and so improve chronic wound care.

Based on our observation on day 3 post wounding, the inflammation score in plasma-treated and control groups was approximately the same. It showed that cold plasma treatment didn’t have any effects on the early stage of inflammation. On days 7, 14 and 21 after wound creation, the inflammation score in the plasma-treated was fewer than in the control. This observation suggested that cold plasma caused acceleration in the late phase of inflammation. Similar results have been reported previously by Tipa RS *et al*.^52^, and Nasruddin *et al*.^18^. These reports stated that this event may be related to the proliferative effect of cold plasma, and the early presence of myofibroblasts, respectively.

On the basis of mechanical data, we showed that cold plasma is efficacious for improving the mechanical strength of repaired tissue on days 14 and 21 after wounding. According to this fact that collagen fibers are responsible for the stiffness and elasticity of the skin tissue, increasing the tensile strength of the tissue can be due to plasma induced collagen synthesis in those tissues, as confirmed by the results of the microscopic evaluation. In addition, there is a strong correlation between increasing the mechanical strength with the wound closure and increasing of tissue tolerance versus rupture.

The present study indicates that wound healing phases were promoted by cold plasma treatment and the wound closure rate was significantly increased from day 7 to 21. Because an increase in wound closure rate was observed, it can be suggested that plasma exposer can inhibit the re-opening of sores, which, in turn, can have therapeutic value in clinical studies. In line with this results, Jacofsky *et al*. reported that helium cold plasma treatment accelerated the wound healing rate in a diabetic murine model^19^. In addition, it was recently demonstrated that cold plasma treatment had a positive effect and support the healing process of chronic wounds in clinical trials^17,34,36^. However, there were no mechanical data in this report. This is the first report describing such findings supported by the mechanical data.

In conclusion, this research demonstrates that cold plasma jet provided an improvement in terms of accelerating the wound healing through promoting of re-epithelialization, wound closure, the late phase of inflammation, and improving strength and rate of maturity in tissue repair. Our results suggest a possible treatment effect of cold plasma for wound healing in pressure ulcers in the future.

## Materials and Methods

### Ethics statement

In this study 45 adult male Wistar rats weighing (250 ± 50 gr) were caged individually under standard laboratory conditions (room temperature, atmospheric pressure, relative humidity: 30 ± 10%, 12 hour light-dark cycle) with free access to food and water. All of the animal experiments and procedures were approved by Biomedical Ethics Committee of Shahid Beheshti University of Medical Sciences (IR.SBMU.RETECH.REC, ethic no. 1396.28). All surgeries were done under deep anesthesia in accordance with the animal ethical guidelines of Shahid Beheshti University of medical science, and all efforts were made to minimize suffering.

### Plasma jet system

**Figure 9:**
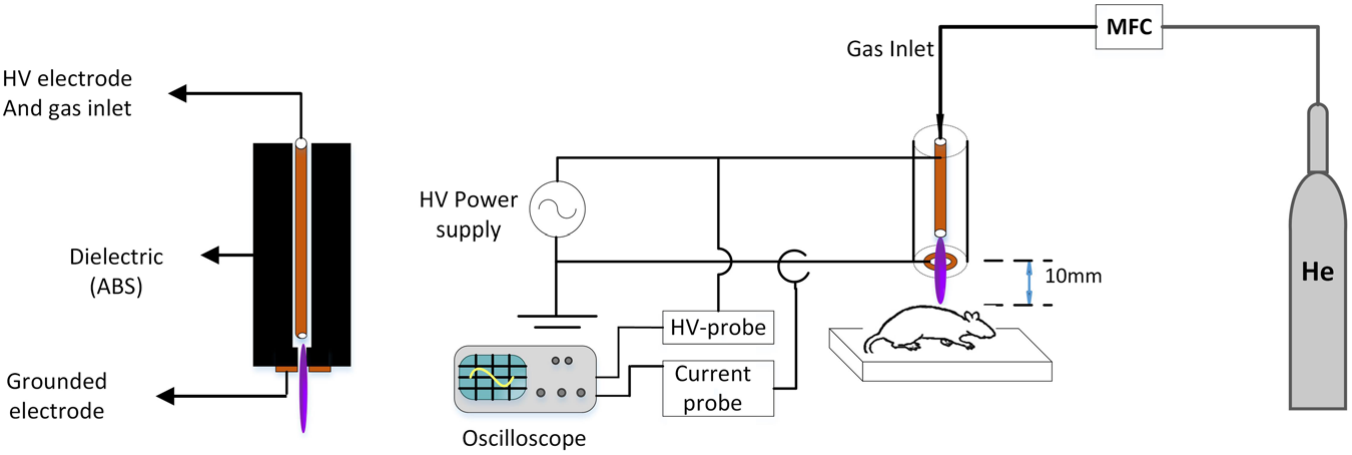
Schematic of experimental set-up and side view of plasma jet.

The plasma jet system used in this study is shown schematically in Fig. 9, which is mainly composed of a copper tube as the central electrode, a copper ring as the ground electrode and a cylinder of Acrylonitrile Butadiene Styrene (ABS), as the dielectric between the electrodes. An AC high-voltage, with a pick-to-pick voltage of 5 kV and frequency of 25 kHz, was applied to the central electrode when helium gas (99.999% purity) at a flow rate set to 4 standard liters per minute (slpm) was injected from one end of the copper tube. The carrier gas flow was controlled using a mass flow controller (APEX, AX-MC-5SLPM-D). A high-voltage probe (Tekteronix, P6015A, 1:1000), a current probe (TCP-202), and also an oscilloscope (Tekteronix, DPO3012) were used to characterization and analysis the electrical behavior of plasma.

### Wound model and treatment protocol

**Figure 10:**
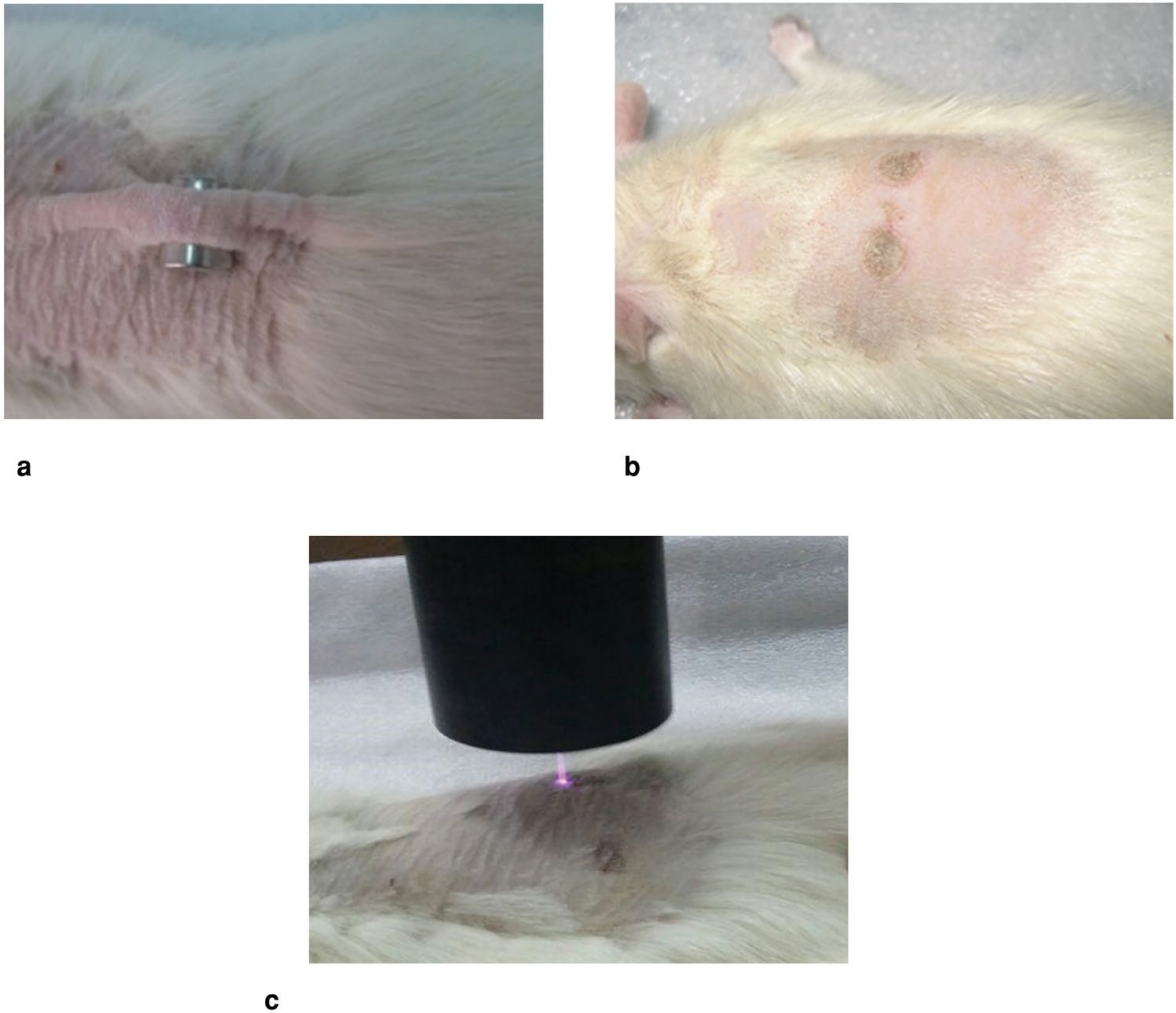
(**a**) Creation of experimental pressure sore in the rat’s skin using magnet. (**b**) Circular pressure ulcers created on skin. (**c**) Treating wounds with cold plasma radiation.

In this study, we used the pressure ulcer model reported by Stadler *et al*. (2004)^53^. After weighing the animals and general anesthesia by injection a mixture of xylazine hydrochloride (10 mg/kg) and ketamine hydrochloride (100 mg/kg), the hair on the dorsum was shaved and the area cleaned with 70% alcohol. As shown in Fig. 10(a), the skin was gently pulled up and placed between two circular permanent magnets that had 10 mm diameter and 5 mm thick, with 4000 G magnetic force. Magnets were remained on the skin for 8 hours and 72 hours after ending pressure, 2 circular pressure ulcers with nearly the same diameter and area, were made in each animal (Fig. 10(b)).

In this study, animals were studied in three groups: plasma-treated, control and normal. The wounded animals were randomly classified into plasma-treated and control groups (20 rats in each group), and were compared to the normal group (5 rats without skin wounds) in the mechanical parameters. Histological and mechanical parameters of tissue were assessed for wounded animals at 4 time points during healing period (totally 5 animals or 10 wounded tissue were allocated in each of control and plasma-treated groups at each time point).

In the plasma-treated group, wounds were treated 3 times daily for 5 days. Each time, plasma radiation lasted 60 s with 30 s interruptions. The position of the wound surface was about 10 mm under the nozzle tip, as shown in Fig. 10(c).

### Macroscopic evaluation

The day when wounds were appeared (72 hours after ending pressure), was considered as Day 0, and the wound healing process was evaluated morphologically, from Day 0 to Day 21 after wounding. Wounds were photographed on Days 0, 3, 7, 14 and 21 after wounding by a digital camera, and wound surface area was calculated using ImageJ 1.49 v image analysis software. The wound closure rate was calculated by

$$ \% \,wound\,closure=[1-(\frac{wound\,area\,{t}_{n}\,}{wound\,area\,{t}_{0}})]\times 100$$

### Histological analysis

Following euthanasia with chloroform inhalation, on days 3, 7, 14 and 21 post-wounding the wounds and the surrounding normal skin, were excised and fixed in neutral buffered 10% formalin solution. 3 samples from each of control and plasma-treated groups were dehydrated in an alcohol series, cleaned in xylene and embedded in paraffin then 5-μm sections were prepared for general staining using hematoxylin-eosin (H&E) and specific staining using Masson’s trichrome staining. Stained tissues were evaluated under a light microscope (Euromex, FE.2025) with attached digital camera system (euromex, CMEXDC.5000) and scored using Ehrlich-Hunt numerical scale^54^. Inflammation, the intensity of fibrosis (collagen synthesis) and angiogenesis were graded as 0 for absence, 1 for minimal, 2 for mild, and 3 for severe. Epithelialization was also scored as 0 for absence, 1 for focal presence, 2 for thin-complete surface, and 3 for thick-complete surface. Finally, the average data was reported.

### Mechanical assay

On Days 3, 7, 14 and 21 after wounding, strips of skin (60 mm × 20 mm) included the wounds and the surrounding normal skin, were excised from sacrificed animals (7 specimens from each of control and plasma-treated groups). The sample thickness was measured by a digital caliper. The specimens were kept moistened in 0.9% NaCl solution at −20°c until the test time^55,56^. In this test, skin strips were fixed between two clamps of a material testing machine (Zwick-Roll, Z25-ph1F, Germany) and stretched by uniaxial tensile test at a constant speed of 10 mm/min until the skin strips were ruptured. Stress-Strain curves were recorded and the related mechanical parameters [maximum force (F_max_-N), maximum stress (N/mm^2^), work-up to maximum force (W up to F_max_-Nmm) and elastic stiffness (E-Modulus-N/mm^2^)] were calculated by a computer connected to the material testing instrument. The mechanical results were analyzed to evaluate plasma effects on mechanical parameters of tissue.

The maximum force (N) was obtained directly from the curve and represents the maximum tolerance of the tissue against rupture or maximum tensile force applied to the specimen to rupture it. Maximum stress (N/mm^2^) was calculated by dividing the maximum force over the cross-sectional area of the specimen. W up to F_max_ (Nmm) was measured by calculating the area under the curve and represents energy absorption by the tissue under the tensile force applying. E-Modulus (N/mm^2^) was obtained from the slope of the linear part of the Stress-Strain curve and represents the elastic stiffness of tissue that is a very important parameter in the evaluation of wound healing process^57,58^.

### Optical emission spectroscopy

Optical emission spectroscopy (OES), is an appropriate noninvasive method to determine different reactive species produced in plasma. OES was done using an optical fiber connected to the spectrometer (AVANTES, AvaSpec 3648).

### Thermal evaluation

The temperature of plasma is a very important parameter and its thermal effect on living tissue and safety must be evaluated. So prior to plasma application on the wound it was tested on normal skin of the anesthetized mouse. The temperature of the skin was measured with a non-contact infra-red digital camera (FLIR E4) in different voltages during 1 min plasma treatment (about 6 images were taken during 1 min).

The rotational and vibrational temperatures can be easily estimated by fitting the experimental and simulated spectra. Specair software and wavelengths 370–381 nm (nitrogen second positive system transition) were used to simulate and compare the spectra.

### Statistical analysis

Statistical analysis was performed using SPSS 19.0. Normal distribution of data was analyzed using Shapiro-Wilk test (p > 0.05). Therefore repeated-measures analysis (to compare data in each group) and independent t-test (to compare data between two groups) were used for wound area decrease analysis. Mechanical parameters were compared among groups by ANOVA followed by the Tukey test. For comparison of the groups in terms of collagen synthesis, angiogenesis, inflammation, and epithelialization, Mann-Whitney-U test was used because variables were categorical data. Differences with p < 0.05 were considered statistically significant.
